# Psychopathological characterization of modern-type depression in subjects with Internet Gaming Disorder

**DOI:** 10.1192/j.eurpsy.2023.2126

**Published:** 2023-07-19

**Authors:** L. Orsolini, G. Longo, C. Ambrosi, U. Volpe

**Affiliations:** University Psychiatric Clinic, Department of Clinical Neuroscience/DIMSC, Università Politecnica delle Marche, Ancona, Italy

## Abstract

**Introduction:**

In recent years, more evidence is emerging in favor of a new form of depression, aka “Modern-Type Depression” (MTD). It has also been theorized that MTD may have multiple relationships with other psychiatric disorders, including techno-addictions.

**Objectives:**

Our study aims at clinically characterizing subjects with MTD in a sample of individuals affected with Internet Gaming Disorder (IGD).

**Methods:**

1,157 subjects were recruited from a sample of Italian young people (aged 18-35), and selected only if they declared to be video game players (48.6%, n=542). Video game players filled out the 22-item Tarumi’s Modern-Type Depression Trait Scale (TACS-22), Motives for Online Gaming Questionnaire (MOGQ), Internet Gaming Disorder Scale-Short-Form (IGDS9-SF), Problematic Online Gaming Questionnaire (POGQ), Multidimensional State Boredom Scale (MSBS), Symptom Checklist-90 (SCL-90). Subjects were classified as IGD+/IGD- and MTD+/MTD-. Descriptive analysis, Mann-Whitney’s U-test for independent data and Chi-square tests were carried out.

**Results:**

60.5% (n=328) of the sample were male. 21.7% (n=118) were positive to MTD. MTD subjects reported significantly higher scores at IGDS9-SF (p<0.001), POGQ (p<0.001), MOGQ (p=0.003), MSBS (p<0.001). Significant higher scores were found at the MOGQ subscales “reality avoidance” (p<0.001), “coping” (p=0.001), and “fantasy” (p<0.001) and at the SCL-90 subscales “interpersonal sensitivity” (p<0.001), “phobic anxiety” (p<0.001), and “psychoticism” (p<0.001).

**Conclusions:**

MTD displayed a strong association with technopathies, particularly IGD. Therefore, further studies should evaluate whether MTD could represent a predictor to IGD onset and/or maintenance and adequately address this aspect from a preventive and treatment perspective.

**Disclosure of Interest:**

None Declared

